# On Modern Progress in Ophthalmic Medicine and Surgery

**Published:** 1894-01-13

**Authors:** Robert Brudenell Carter

**Affiliations:** Consulting Ophthalmic Surgeon to St. George's Hospital


### ON MODERN PROGRESS IN OPHTHALMIC MEDICINE AND SURGERY.

By ROBERT BRUDENELL CARTER, F.R.C.S., Consulting Ophthalmic Surgeon to St. George's Hospital.

BEFORE proceeding to describe the indirect effect of the iridectomy controversy, and of the state of things which it disclosed, in assisting to bring about improvements in medical education, it may be well to carry the history of the operation itself down to the present time. For several years it continued where it was left by Mr. Bowman and his immediate coadjutors; that is to say, as a remedy of known efficacy for increased ocular tension, whether this were primary, as in glaucoma, or secondary to some inflammatory affection by which the quantity of fluid within the eyeball had been increased. It was soon found that the full advantages of the operation could only be obtained when it was performed in a particular manner; the chief points requiring attention being that the external incision should be made as far from the margin of the cornea as was compatible with carrying the knife into the anterior chamber in front of the iris, that the wound should be of sufficient length, and that the whole width of the iris should be removed from the pupillary to the ciliary margin. If the incision were made into the true cornea, and if the so-called iridectomy consisted merely of cutting a notch out of the pupillary border, little or no permanent benefit could be secured. In many cases of advanced glaucoma, in which the pupil was dilated and the iris reduced to a narrow ring, and in which the anterior chamber was almost abolished by the projection forward of the lens, pushed by the increased quantity of fluid behind it, the fulfilment of the required conditions was very difficult; and it is probable that some at least of the surgeons who attempted the operation did not succeed in accomplishing all that they desired. Sometimes the knife entered behind the iris; sometimes the lens was wounded, and traumatic cataract produced; sometimes it was not found practicable to seize and draw out the narrow iris, and hence the amount removed was insufficient. Difficulties thus arising gradually disappeared as exact information concerning the essential points of the operation were diffused abroad; and iridectomy was securely placed very much in its present position as a means of treatment before the time came when the reason of its beneficial action could be explained.

The first step in this direction was made by the discovery that an attack of acute glaucoma would sometimes follow the application of atropine to the eye for the purpose of dilating the pupil for opthalmoscopic examination. Such dilatation is now very seldom employed by practised observers; but it was once employed extensively, and under the belief that no evil could be produced by it except a temporary disturbance of the accommodation. The earliest recorded instances of atropine glaucoma were received with some incredulity, or were looked upon as mere coincidences; but such coincidences soon became too numerous to be neglected. The existence of an actual connection between the tension of the eye and the diameter of the pupil was also proved by the discovery that the preparations of Calabar bean tend definitely to diminish tension; and these and other related facts were in time so far worked out, chiefly by the researches of Mr. Priestley Smith, as ultimately to afford a fairly clear notion of the pathology of glaucoma, which, notwithstanding certain gaps in our knowledge, may now be expressed somewhat in the following way.

The normal fulness or tension of the eye, by which the necessary tightness of its tunics is preserved, and the curvature of the retina is maintained, is a result of the continued internal secretion of fluid, chiefly by the ciliary processes, and of its continued removal by exhalation or filtration through the tunics. This filtration mostly occurs in the zone of tissue which immediately intervenes between the true cornea and the true sclera, a zone pierced by a circular (Schlemm's) canal, which contains a plexus of veins. This zone, now usually called the "filtration area," is in front of the peripheral attachment of the iris, and the porosity or laxity of tissue of the area permits any superfluous fluid within the eye to enter Schlemm's canal, where it passes into the veins and is carried into the general current of the circulation. The necessary transudation occurs with the greatest freedom when the pupil is contracted, because then the iris is thinned out, and is drawn away from the angle of entrance to the filtration area, an angle formed between the anterior surface of the iris and the posterior surface of the cornea. The fluid contained in the anterior chamber can then obtain free access to the filtration area; and, as this fluid passes out of the eye, its place is taken by fresh secretion which passes forward into the anterior chamber through the pupil. When, on the other hand, the pupil is dilated, the circle of iris tissue around the pupil is thickened, and tends to fill up the angle, and thus to obstruct the access of fluid to the filtration area. In this way, in an eye predisposed to glaucoma, the mere dilatation of the pupil by atropine may suffice to bring on an acute attack. Even when this does not occur, a degree of dilatation which impedes the exit of fluid from the eye, but which rather facilites its passage into the anterior chamber through the pupil, permits the incoming current to push the pupillary margin of the iris forward, and to apply its peripheral zone to the filtration area somewhat after the manner of a valve, so as to arrest transudation almost entirely. In cases of chronic glaucoma, the peripheral zone of iris will sometimes contract inflammatory adhesion to the inner surface of the filtration area, and then the chronic affection rapidly passes into one that is acute. The physiological state of the iris, in relation to the thickness of its tissue, and to the position of its peripheral zone, is therefore the chief (and an almost mechanical) factor in the production of glaucoma, nearly all the symptoms of which admit of a simple and rational explanation. When once the mechanism of increased tension is understood, the effect of iridectomy in relieving it becomes self-evident. The incision evacuates the whole of the aqueous humour; and, when this is re-secreted and the wound healed, the part of the filtration area corresponding to the periphery of the piece excised is completely freed from the presence of iris, and from any impediment to transudation which it may have caused. It is also probable, when the incision into the eye has been carefully and skilfully made, that the permeability of the filtration area in the neighbourhood of the cicatrix will be increased rather than diminished.

